# Piscine orthoreovirus‐3 is prevalent in wild seatrout (*Salmo trutta* L.) in Norway

**DOI:** 10.1111/jfd.12943

**Published:** 2019-01-18

**Authors:** Åse Helen Garseth, Torfinn Moldal, Siri Kristine Gåsnes, Monika Jankowska Hjortaas, Vegard Pedersen Sollien, Anne‐Gerd Gjevre

**Affiliations:** ^1^ Norwegian Veterinary Institute Oslo Norway; ^2^ Norwegian Food Safety Authority Oslo Norway

**Keywords:** Arctic char (*Salvilinus alpinus* L.), Atlantic salmon (*Salmo salar* L.), landlocked, PRV‐3, trout (*Salmo trutta* L.), wild

## Abstract

In 2017, a PCR‐based survey for Piscine orthoreovirus‐3 (PRV‐3) was conducted in wild anadromous and non‐anadromous salmonids in Norway. In seatrout (anadromous *Salmo trutta* L.), the virus was present in 16.6% of the fish and in 15 of 21 investigated rivers. Four of 221 (1.8%) Atlantic salmon (*Salmo salar* L.) from three of 15 rivers were also PCR‐positive, with Ct‐values indicating low amounts of viral RNA. All anadromous Arctic char (*Salvelinus alpinus* L.) were PCR‐negative. Neither non‐anadromous trout (brown trout) nor landlocked salmon were PRV‐3 positive. Altogether, these findings suggest that in Norway PRV‐3 is more prevalent in the marine environment. In contrast, PRV‐3 is present in areas with intensive inland farming in continental Europe. PRV‐3 genome sequences from Norwegian seatrout grouped together with sequences from rainbow trout (*Oncorhynchus mykiss* Walbaum) in Norway and Coho salmon (*Oncorhynchus kisutch* Walbaum) in Chile. At present, the origin of the virus remains unknown. Nevertheless, the study highlights the value of safeguarding native fish by upholding natural and artificial barriers that hinder introduction and spread, on a local or national scale, of alien fish species and their pathogens. Accordingly, further investigations of freshwater reservoirs and interactions with farmed salmonids are warranted.

## 1 INTRODUCTION

Heart and Skeletal Muscle Inflammation (HSMI) in Atlantic salmon (*Salmo salar* L.) was first recorded in 1999 (Kongtorp, Kjerstad, Kjerstad, Taksdal, Guttvik, & Falk, 2004; Kongtorp, Taksdal, Taksdal, & Lyngoy, 2004) and associated with a piscine orthoreovirus virus (PRV) in 2010 (Palacios et al., 2010). Today, HSMI is one of the most common viral diseases of farmed Atlantic salmon in Norway, causing major economic and biological losses due to mortality, especially in combination with stress and various forms of handling (Hjeltnes, Bang‐Jensen, Bornø, Haukaas, & Walde, 2018). Over the past few years, research efforts and new bioinformatics tools have increased our knowledge about both the virus and the disease. Accordingly, the causal relationship between PRV and HSMI was firmly established in 2017 (Wessel et al., 2017), and new subtypes of PRV have been described and linked to several disease conditions. The association between Erythrocytic inclusion body syndrome (EIBS) in Coho salmon (*Oncorhynchus kisutch *Walbaum) and PRV2 was described in 2016 (Takano et al., 2016), and PRV‐3 has been described in association with HSMI‐like lesions in rainbow trout (*Oncorhynchus mykiss* Walbaum) in Norway and Coho salmon in Chile (Godoy et al., 2016; Olsen, Hjortaas, Hjortaas, Tengs, Hellberg, & Johansen, 2015). The nomenclature used is in line with proposal in Dhamotharan et al. (2018).

In 2013, rainbow trout fingerlings from three different hatcheries in Norway showed disease signs and necropsy findings resembling HSMI in Atlantic salmon. Gross necropsy findings were signs of circulatory disturbances including haemorrhages, ascites and anaemia, and histopathological findings comprised inflammation of the heart and red skeletal muscle and necrosis in the liver. Two of the hatcheries reported low to moderate mortality. The third hatchery reported 0.3% mortality in tanks without clinical signs, and up to 21% in tanks with diseased fish. Disease and related mortality was reported up to four months after sea transfer. Extended investigations, initiated due to the resemblance with HSMI in Atlantic salmon, led to the identification of a virus with gene sequences sharing approximately 85% identity with PRV in Atlantic salmon and closely related a PRV‐like virus in Coho salmon. Hence, both viruses probably belong to the same group with the suggested designation PRV‐3 (Dhamotharan et al., 2018).

Experimental infection studies have strengthened the association between PRV‐3 and the disease signs seen in rainbow trout (Hauge et al., 2017). The infection transmitted to all of rainbow trout cohabitants that also developed disease symptoms. The virus seemed less adapted to Atlantic salmon, as less than 50% of the cohabitants were infected 8–16 weeks post‐challenge and experienced only minor lesions.

Epidemiological investigations and PCR‐based surveillance programmes show that PRV‐3 is present at all levels of the marine rainbow trout production cycle, including broodfish, hatcheries and after sea transfer (Olsen et al., 2015). PRV‐3 has also been detected in farmed rainbow trout in Germany (Adamek et al., 2018) and associated with disease in this species in Denmark and Scotland (Dhamotharan et al., 2018). In brown trout, the virus has been detected in Italy (Dhamotharan et al., 2018) and France (Bigarre, 2016). In the latter case, mortalities due to a PRV‐3 and infectious pancreatic necrosis virus (IPNV) co‐infection were recorded in juvenile farmed brown trout (*Salmo trutta* L.). Recently, proliferative darkening syndrome (PDS), a severe disease causing die‐off of wild brown trout (*Salmo trutta fario*) in pre‐alpine river systems in Southern Germany, Austria and Switzerland has been associated with PRV‐3.

The Norwegian Food Safety Authority annually conducts health monitoring in wild salmonids. In 2017, the objective of the programme was to investigate the occurrence of PRV‐3 in wild salmonids. Here we report the first detection of PRV‐3 in wild seatrout (*Salmo trutta* L).

## 2 MATERIALS AND METHODS

### 2.1 Study sample

**Figure 1:**
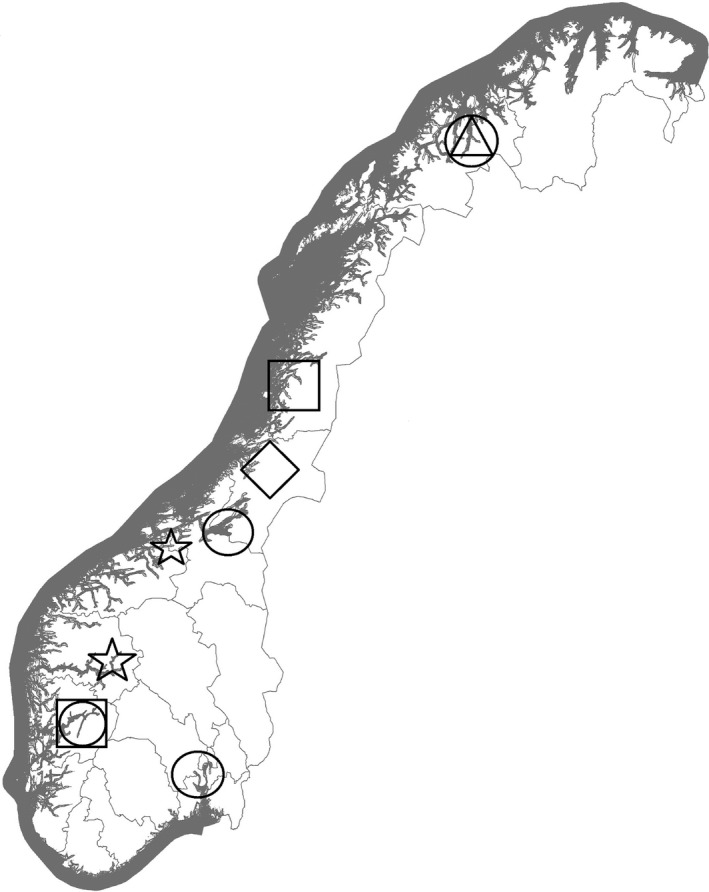
Map of Norway indicating the geographical locations where samples from landlocked salmon (diamond), anadromous salmon (circles), seatrout (squares), Arctic char (triangles) and brown trout (stars) were obtained. The northernmost square indicates the Vefsn region, and the southernmost square indicates the Hardanger region

A cross‐sectional study designed to investigate the occurrence of PRV‐3 in wild adult anadromous and non‐anadromous salmonids in Norwegian watercourses was conducted (Table 1 and Figure 1).

**Table 1:** Overview of study sample including species, anadromousy, year and number of counties, watercourses and fish

Species	Counties	Watercourses	Fish	Years
Anadromous				
Atlantic salmon *Salmo salar* L.	4	15	221	2016
Seatrout *Salmo trutta* L.	2	21	265	2011, 2016, 2017
Arctic char *Salvelinus alpinus* L.	1	2	11	2016
Non‐anadromous				
Landlocked salmon *Salmo salar* L.	1	1	40	2015
Brown trout *Salmo trutta* L.	2	3	79	2010, 2016

The source material comprised wild‐caught broodfish from stock enhancement hatcheries and the Genebank for wild Atlantic salmon (http://www.miljodirektoratet.no/old/dirnat/rapporter/673/rapport.pdf). Broodfish from the same river were kept together in freshwater tanks from a few days up to 6–7 weeks before stripping. Additional samples from seatrout were obtained during rotenone treatment, a measure issued by the Norwegian Environment Agency to control the parasite *Gyrodactylus salaris* in the rivers Hundøla, Vefsna, Fusta and Drevja in 2011 (Anonymous, 2014). Brown trout were sampled from one lake in the County of Trøndelag (2010) and two lakes in the County of Sogn & Fjordane (2016). All Atlantic salmon were classified as wild based on scale reading (Antere & Ikonen, 1983; Fiske, Lund, Lund, & Hansen, 2004; Lund & Hansen, 1991). Figure 1 displays the different regions of Norway represented in the study.

Samples from 2010 to 2016 consisted of heart and kidney tissue fixed in RNAlater^TM^ and were sent frozen to the Norwegian Veterinary Institute (NVI). Samples collected in 2017 consisted of kidney tissue fixed in RNAlater^™^ sent to PatoGen AS for PCR‐analyses.

### 2.2 Real‐time RT‐PCR and sequencing

At NVI, real‐time RT‐PCR for detection of a 121 bp fragment of the sigma 3 protein gene of PRV‐3 was performed using forward primer 5’‐TCG‐TGG‐TTC‐CAA‐TGA‐CAG‐3’, reverse primer 5’‐CCA‐ACC‐ACTAAA‐ACC‐GAG‐3’ and probe 6‐FAM‐5’‐ACG‐CCT‐TAG‐AGA‐CAA‐CAT‐GCG‐AAG‐3’BHQ‐1 and conditions previously described by Olsen et al. (2015). Samples with Ct‐values <40 are reported as positive. Kidney samples collected in 2017 were analysed by PatoGen AS (http://www.patogen.no) and reported as positive if Ct‐values are <37.

From each of the two regions, the two seatrout samples with lowest Ct‐values were selected for verification of positive real‐time RT‐PCR findings by conventional RT‐PCR with forward primer 5’‐GAC‐CAA‐CAT‐AAC‐GTT‐TCA‐GGC‐3’ and reverse primer 5’‐ATC‐CAA‐CCA‐CTA‐AAA‐CCG‐AGA‐3’ amplifying a 423 bp fragment of the sigma 3 protein gene followed by Sanger sequencing and conditions as previously described (Olsen et al., 2015). The obtained sequences were analysed in Sequencher^®^ version 5.4.5 sequence analysis software (Gene Codes Corporation) followed by BLAST search against the GenBank database. All PCR‐positive salmon, all PCR‐positive seatrout from Vefsn and in addition the two seatrout samples selected for sequencing from the Hardanger region were examined for the presence of PRV‐1 (Garseth, Ekrem, Ekrem, & Biering, 2013).

## 3 RESULTS

### 3.1 Real‐time RT‐PCR and sequencing

The PCR‐based screening showed that PRV‐3 was present in wild seatrout (Table 2). Three of 60 seatrout from the Vefsn region (Nordland County, 2011), 31 of 137 samples from Hardanger (Hordaland County, 2016) and finally ten of 68 samples from Hardanger (2017) were positive in real‐time RT‐PCR for detection of PRV‐3. Altogether, 44 virus‐positive seatrout were detected in 15 of 21 rivers and in both regions included in the study. Ct‐values ranged from 22.5 to 39.6. The PCR‐results of two samples from Vefsn and two samples from Hardanger 2016 were verified by repeated real‐time RT‐PCR performed on re‐extracted nucleic acids. Subsequent sequencing and BLAST search against the GenBank database showed that all four samples from seatrout had 99%–100% identity with PRV‐3 in rainbow trout (GenBank accession number LN680851). One sequence from each region was submitted to GenBank and assigned accession numbers MK061361 (River Hundåla/Vefsn) and MK061362 (River Sima/Hardanger). None of the examined samples from seatrout were positive for PRV‐1.

**Table 2:** Overview of results from real‐time PCR‐analyses for PRV‐3 in seatrout (anadromous trout *Salmo trutta* L.). PCR results are presented as the proportion of test‐positive among tested (test‐positive/Number tested).

Region	River	2011	2016	2017	Overall	Ct‐value (range)
Nordland	Drevja	0/14			0/14	
	Fusta	0/6			0/6	
	Hundåla	2/30			2/30	22.5–29.8
	Vefsna	1/10			1/10	31.6
Hardanger	Austrepoll		2/13	1/12	3/25	32.0–35.2
	Fjæra		0/3		0/3	
	Granvin		4/12		4/12	29.0–36.2
	Jondal		4/9	0/5	4/14	28.9–37.6
	Kinso			0/2	0/2	
	Mundheim			1/9	1/9	36.1
	Omvik		5/9		5/9	33.0–36.3
	Opo		0/1		0/1	
	Osa		4/9	3/9	7/18	31.6–34.1
	Rosendal		2/11	1/3	3/14	26.8–31.0
	Sima		2/15	3/20	5/35	28.6–35.4
	Steinsdal		4/25	0/6	4/31	31.7–39.6
	Strandadal		0/8		0/8	
	Uskedal		1/9		1/9	36.3
	Ådland		0/8	1/2	1/10	32.8
	Ænes		2/4		2/4	29.5–31.6
	Øyreselva		1/1		1/1	33.4
Overall range of Ct‐values						22.5–39.6
Fish positive/tested		3/60	31/137	10/68	44/265	
Rivers positive/tested		2/4	11/15	6/9	15/21	

In wild Atlantic salmon, PRV‐3 was detected in four of 221 samples with Ct‐values close to the limit of detection (34.6–40). These results could not be confirmed by sequencing due to low amount of viral RNA. Positive samples originated from three rivers in the Hardanger region. Two of these were also positive for PRV‐1.

## 4 DISCUSSION

### 4.1 PRV‐3 in wild seatrout

In total, PRV‐3 was present in seatrout from 15 of 21 investigated rivers and in both the northern and southern region (Figure 1). This result is independent of the two cut‐offs (<40 or <37), since all the rivers had results below Ct 37. The possible influence of the difference in sampling tissues (heart vs. kidney) on the prevalence of PRV‐3 is thus far unknown. Overall, the per‐river sample sizes in this study were low (mean 12.6, range 1–35). The sample size range of the six virus‐negative rivers was 1–14 (mean 5.7). Consequently, the minimum detectable prevalence in each of these rivers is quite high, indicating that the study underestimates the actual occurrence of PRV‐3 in seatrout (Cameron & Baldock, 1998). Accordingly, this study strongly suggests that PRV‐3 is a common virus in seatrout in both regions.

The prevalence of PRV‐3 is different between the two regions, with 20% virus‐positive in Hardanger and 5% in the Vefsn region. This may reflect actual differences between regions or confounding factors. There is an inherent bias caused by the possibility of virus transmission during cohabitation in tanks in the material from Hardanger. The two regions also differ in year of sampling, 2016/2017 versus 2011, but it is probably more important that seatrout from the Vefsn region comprise a sample of adult seatrout dead due to rotenone exposure in august 2011 (Anonymous, 2014), while all seatrout from Hardanger are mature broodfish. Hormonal changes and stressful handling in Hardanger may have compromised the immune system and rendered the broodfish more susceptible to viral infections (Pickering, 1986; Pickering & Pottinger, 1989). Nevertheless, the virus is present in 13 of 17 rivers (76.5%) in Hardanger and in two of four rivers in the Vefsn region, hence the conclusion that PRV‐3 is common in seatrout sustains. Screening of all life stages of seatrout and in new regions would add more knowledge.

None of the PRV‐3 positive seatrout selected for PRV‐1 analyses were PCR‐positive. Previous studies have shown that approximately 1%–3% of seatrout are PRV‐1‐positive (Garseth, Fritsvold, Opheim, Skjerve, & Biering, 2012; Madhun et al., 2016), but only one case had viral loads sufficiently high to enable sequencing and phylogenetic analyses. In that case, the sequence grouped together with PRV‐1 from wild and farmed salmon (Garseth et al., 2013). For a specific pathogen and a specific host‐population, the prevalence will reflect the efficacy of pathogen transmission, susceptibility of the host and duration of the infection, which in turn depends on the virulence of the pathogen and the immunization rate of the host (Begon, Harper, & Townsend, 1990). Transmission trials have shown that PRV‐1 is less adapted to seatrout than to Atlantic salmon (Grefsrud et al., 2018). Consequently, this survey has revealed a much higher prevalence and viral load of PRV‐3 in seatrout than previously recorded for PRV‐1, and furthermore, that the PRV‐3 sequences group together with sequences from rainbow trout and Coho salmon in Chile. According to Dhamotharan et al., 2018, two clades of PRV‐3 are present. Unfortunately, we were not able to assign sequences to specific clades.

### 4.2 PRV‐3 in wild Atlantic salmon

Transmission trials have shown that PRV‐3 is less adapted to Atlantic salmon than rainbow trout (Hauge et al., 2017). Cohabitation in tanks with infected seatrout could be a bias in the study. However, none of the PRV‐3 positive salmon were kept in tanks with PRV‐3 positive seatrout.

### 4.3 Reservoirs and interaction between farmed and wild salmonids

None of the 11 anadromous Arctic char were virus‐positive. However, the sample size was low and only suitable for detecting highly prevalent pathogens (Cameron & Baldock, 1998).

Altogether, 79 brown trout from three non‐anadromous lakes were tested without detecting PRV‐3. Similarly, the virus was absent in 40 non‐anadromous (landlocked) salmon (Table 1). This could mean that salmonids in relatively naïve non‐anadromous watercourses are of minor importance as a reservoir. PRV‐3 is nevertheless common in seatrout from anadromous watercourses, meaning that rainbow trout farms that use these water sources are at risk.

In Norway, farming of rainbow trout takes place as two nearly separate production lines: the freshwater‐based small‐scale inland aquaculture (based on of roe from the marine production), and the large‐scale marine production along the coast, where juvenile stages are produced in freshwater. In the marine aquaculture of rainbow trout, PRV‐3 is present at all stages of the production cycle, including hatcheries that do not use sea water in their production (Olsen et al., 2015). At any given time, there are approximately 20 million (35,000 metric tonnes) of sea‐farmed rainbow trout present in Norwegian coastal waters (Directorate of fisheries; Statistics for aquaculture 2018). The high number and biomass of susceptible and infected farmed hosts represents a considerable potential for virus propagation. Accordingly, seatrout that are exposed to effluents from hatcheries or infected coastal waters are at risk. Both counties represented in this study have a steady production of rainbow trout in the sea (Directorate of fisheries; Statistics for aquaculture 2018), and sequences obtained from wild seatrout had 99%–100% identity with PRV‐3 in rainbow trout. Accordingly, virus transmission between seatrout and farmed and escaped rainbow trout is likely to occur.

The freshwater‐based inland aquaculture takes place adjacent important native freshwater fish stocks, including stocks of brown trout. Thus far, the occurrence of PRV‐3 in inland aquaculture of rainbow trout and wild stocks in these areas has not been studied. Reports from continental Europe provide evidence that PRV‐3 is present in inland farming of rainbow trout and brown trout (Adamek et al., 2018; Bigarre, 2016; Dhamotharan et al., 2018) elaborate on the sequence identity of PRV‐3 found in seatrout. Further investigations of freshwater resident wild and farmed populations in Norway are therefore necessary.

In a broader perspective, it is interesting to discuss PRV‐3 encountered in Norwegian seatrout and rainbow trout and the similarities with PRV‐3 in Chilean Coho salmon. Historically, rainbow trout, but also European *Salmo trutta* have been introduced and released in Chile and could thus have introduced the virus. On the other hand, global trade of fish and eggs is an ongoing activity and will always represent a risk of introduction of both alien fish species and their pathogens. The results from this study therefore highlight the value of safeguarding native fish species by upholding natural and artificial barriers that hinder introduction and spread of alien fish species and their pathogens.

## 5 CONCLUSIONS

Piscine orthoreovirus subtype 3 (PRV‐3) is a common virus in seatrout in Norway. Compared to rainbow trout, Atlantic salmon are less susceptible to the virus, which may explain the low prevalence and viral loads recorded in wild specimen in this study. The absence of PRV‐3 in both non‐anadromous *Salmo trutta* (brown trout) and *Salmo salar* (landlocked salmon) indicates that the virus may be linked to the marine environment in Norway. Olsen et al., (2015) referred to PRV‐3 as a rainbow trout associated virus. This study strongly supports an association with seatrout.

## CONFLICT OF INTERESTS

No conflict of interest has been identified for any of the authors.

## References

[jfd12943-bib-0001] Adamek, M. , Hellmann, J. , Flamm, A. , Teitge, F. , Vendramin, N. , Fey, D. , … Steinhagen, D. (2018). Detection of piscine orthoreoviruses (PRV‐1 and PRV‐3) in Atlantic salmon and rainbow trout farmed in Germany. Transbound Emerging Diseases. 10.1111/tbed.13018. 30230250

[jfd12943-bib-0002] Anonymous (2014). Bekjempelse av Gyrodactylus salaris i Vefsnaregionen InStensli J. H., & Bardal H. (Eds.), Veterinærinstituttets rapportserie 2014:2, Oslo: Norwegian Veterinary Institute Retrieved from https://www.vetinst.no/rapporter-og-publikasjoner/rapporter/2014/bekjempelse-av-gyrodactylus-salaris-i-vefsnaregionen (Accesssed 14 October 2018, In Norwegian).

[jfd12943-bib-0003] Antere, I. , & Ikonen, E. (1983). A method of distinguishing wild salmon from those originating from fish farms on the basis of scale structure. Ices Journal of Marine Science, 26, 1–11.

[jfd12943-bib-0004] Begon, M. , Harper, J. L. , & , (1990). Ecology: individuals, populations and communities. Oxford, England: Blackwell Scientific Publications.

[jfd12943-bib-0005] Bigarre, L. (2016). First detection of Piscine reovirus in France. Oral presentation at PD‐Trination meeting. 12–13 Octobre 2016, Aberdeen, Scotland Retrieved from http://trination.org/wp-content/uploads/2016/12/2016_D2_1_Bigarre_PRV-France.pdf

[jfd12943-bib-0006] Cameron, A. R. , & Baldock, F. R. (1998). A new probability formula for surveys to substantiate freedom from disease. Preventive Veterinary Medicine, 34, 1–17. 10.1016/S0167-5877(97)00081-0. 9541947

[jfd12943-bib-0007] Dhamotharan, K. , Vendramin, N. , Markussen, T. , Wessel, Ø. , Cuenca, A. , Nyman, I. , … Rimstad, E. (2018). Molecular and antigenic characterization of piscine orthoreovirus (PRV) from rainbow trout (*Oncorhynchus mykiss*). Viruses, 10(4), 170 10.3390/v10040170. PMC5923464 29614838

[jfd12943-bib-0008] Fiske, P. , Lund, R. A. , & Hansen, L. P. (2004). Identifying fish farm escapees In Cadrin S., Friedland K., & Waldman J. (Eds.), Stock identification methods (pp. 659–680). Amsterdam: Elsevier.

[jfd12943-bib-0009] Garseth, A. H. , Ekrem, T. , & Biering, E. (2013). Phylogenetic evidence of long distance dispersal and transmission of piscine reovirus (prv) between farmed and wild Atlantic Salmon. PLoS ONE, 8(12), e82202 10.1371/journal.pone.0082202. 24349221 PMC3859594

[jfd12943-bib-0010] Garseth, A. H. , Fritsvold, C. , Opheim, M. , Skjerve, E. , & Biering, E. (2012). Piscine reovirus (PRV) in wild Atlantic salmon, *Salmo salar* L., and sea‐trout, *Salmo trutta* L., in Norway. Journal of Fish Diseases, 36(5), 483–493. 10.1111/j.1365-2761.2012.01450.x. 23167652

[jfd12943-bib-0011] Godoy, M. G. , Kibenge, M. J. , Wang, Y. , Suarez, R. , Leiva, C. , Vallejos, F. , & Kibenge, F. S. (2016). First description of clinical presentation of piscine orthoreovirus (PRV) infections in salmonid aquaculture in Chile and identification of a second genotype (Genotype II) of PRV. Virology Journal, 13, 98 10.1186/s12985-016-0554-y. 27296722 PMC4906990

[jfd12943-bib-0012] Grefsrud, E. S. , Glover, K. , Grøsvik, B. E. , Husa, V. , Karlsen, Ø. , Kristiansen, T. , … Svåsand, T. (Eds.) (2018). Risikorapport norsk fiskeoppdrett 2018. Fisken og havet, særnr. 1–2018. Bergen: Institute of Marine Research Retrieved from: https://www.imr.no/filarkiv/2018/02/risikorapport_2018.pdf/nn-no (Accessed 14 October 2018, In Norwegian).

[jfd12943-bib-0013] Hauge, H. , Vendramin, N. , Taksdal, T. , Olsen, A. B. , Wessel, O. , Mikkelsen, S. S. , … Dahle, M. K. (2017). Infection experiments with novel Piscine orthoreovirus from rainbow trout (*Oncorhynchus mykiss*) in salmonids. PLoS ONE, 12(7), e0180293 10.1371/journal.pone.0180293. 28678799 PMC5497981

[jfd12943-bib-0014] Hjeltnes, B. , Bang‐Jensen, B. , Bornø, G. , Haukaas, A. , & Walde, C. S. (Eds.) (2018) The Health Situation in Norwegian Aquaculture 2017, Norwegian Veterinary Institute Retrieved from https://www.vetinst.no/rapporter-og-publikasjoner/rapporter/2018/fish-health-report-2017 (Accessed 14 October 2018).

[jfd12943-bib-0015] Kongtorp, R. T. , Kjerstad, A. , Taksdal, T. , Guttvik, A. , & Falk, K. (2004). Heart and skeletal muscle inflammation in Atlantic salmon, *Salmo salar* L.: A new infectious disease. Journal of Fish Diseases, 27(6), 351–358. 10.1111/j.1365-2761.2004.00549.x. 15189375

[jfd12943-bib-0016] Kongtorp, R. T. , Taksdal, T. , & Lyngoy, A. (2004). Pathology of heart and skeletal muscle inflammation (HSMI) in farmed Atlantic salmon Salmo salar. Diseases of Aquatic Organisms, 59(3), 217–224. 10.3354/dao059217. 15264718

[jfd12943-bib-0017] Lund, R. A. , & Hansen, L. P. (1991). Identification of wild and reared Atlantic salmon, Salmo salar L., using scale characters. Aquaculture and Fisheries Management, 22, 499–508. 10.1111/j.1365-2109.1991.tb00763.x.

[jfd12943-bib-0018] Madhun, A. S. , Isachsen, C. H. , Omdal Strandenes, L. M. , Einen, A. C. , Bjørn, P. A. , Nilsen, R. , & … E. (2016). Occurrence of salmonid alphavirus (SAV) and piscine orthoreovirus (PRV) infections in wild sea trout Salmo trutta in Norway. Diseases of Aquatic Organisms, 120, 109–113. 10.3354/dao03009 27409234

[jfd12943-bib-0019] Olsen, A. B. , Hjortaas, M. , Tengs, T. , Hellberg, H. , & Johansen, R. (2015). First description of a new disease in rainbow trout (*Oncorhynchus mykiss* (Walbaum)) similar to heart and skeletal muscle inflammation (HSMI) and detection of a gene sequence related to piscine orthoreovirus (PRV). PLoS ONE, 10(7), e0131638. 26176955 10.1371/journal.pone.0131638 PMC4503464

[jfd12943-bib-0020] Palacios, G. , Lovoll, M. , Tengs, T. , Hornig, M. , Hutchison, S. , Hui, J. , … Lipkin, W. I. (2010). Heart and skeletal muscle inflammation of farmed salmon is associated with infection with a novel reovirus. PLoS ONE, 5(7), e11487 10.1371/journal.pone.0011487. 20634888 PMC2901333

[jfd12943-bib-0021] Pickering, A. D. (1986). Changes in blood cell composition of the brown trout, Salmo salar L., during the spawning season. Journal of Fish Biology, 29, 335–347. 10.1111/j.1095-8649.1986.tb04950.x

[jfd12943-bib-0022] Pickering, A. D. , & Pottinger, T. G. (1989). Stress responses and disease resistance in salmonid fish - effects of chronic elevation of plasma-cortisol. Fish Physiology and Biochemistry, 7, 253–258. 10.1007/BF00004714 24221779

[jfd12943-bib-0023] Takano, T. , Nawata, A. , Sakai, T. , Matsuyama, T. , Ito, T. , Kurita, J. , … Nakayasu, C. (2016). Full‐genome sequencing and confirmation of the causative agent of erythrocytic inclusion body syndrome in Coho Salmon identifies a new type of piscine orthoreovirus. PLoS ONE, 11(10), e0165424 10.1371/journal.pone.0165424. 27788206 PMC5082797

[jfd12943-bib-0024] Wessel, O. , Braaen, S. , Alarcon, M. , Haatveit, H. , Roos, N. , Markussen, T. , … Rimstad, E. (2017). Infection with purified Piscine orthoreovirus demonstrates a causal relationship with heart and skeletal muscle inflammation in Atlantic salmon". PLoS ONE, 12(8), e0183781 10.1371/journal.pone.0183781. 28841684 PMC5571969

